# Complete Genome Sequence of *Bacillus thuringiensis* Bacteriophage BMBtp2

**DOI:** 10.1128/genomeA.00011-12

**Published:** 2013-01-15

**Authors:** Zhaoxia Dong, Donghai Peng, Yueying Wang, Lei Zhu, Lifang Ruan, Ming Sun

**Affiliations:** State Key Laboratory of Agricultural Microbiology, College of Life Science and Technology, Huazhong Agricultural University, Wuhan, China

## Abstract

*Bacillus thuringiensis* is an insect pathogen which has been widely used for biocontrol. During *B. thuringiensis* fermentation, lysogenic bacteriophages cause severe losses of yield. Here, we announce the complete genome sequence of a bacteriophage, BMBtp2, which is induced from *B. thuringiensis* strain YBT-1765, which may be helpful to clarify the mechanism involved in bacteriophage contamination.

## GENOME ANNOUNCEMENT

As an insect pathogen, the Gram-positive bacterium *Bacillus thuringiensis* (Bt) has been used widely for insect control for several decades (1). Bacteriophage contamination causes great losses during Bt fermentation, especially those from the lysogenic bacteriophages. Up to 15 to 30% of the total batches, and sometimes up to 50 to 80%, were damaged by bacteriophages (2). Several methods have been applied to resolve this problem. However, the genomic characterization for regulating the lysogeny and lytic states of Bt bacteriophages is yet unclear.

The temperate bacteriophage BMBtp2 was produced via induction by mitomycin C from *Bacillus thuringiensis* subsp. *tenebrionis* strain YBT-1765 (3). BMBtp2 is a typical tailed bacteriophage with a 54-nm icosahedral head and a 162-nm long noncontractile tail (data not shown), and it belongs to the *Siphoviridae* family according to its morphological characteristics (4). One hundred forty Bt strains were used to examine their sensitivity to BMBtp2, and only six strains were found to be sensitive to BMBtp2 (data not shown).

Bacteriophage morphologies were examined by transmission electronic microscopy (TEM) (Tecnai 10). The genomic DNA of BMBtp2 was prepared by precipitation by ZnCl_2_ (5). The genome was sequenced using Illumina/Solexa GAIIx at the Beijing Genomics Institute (BGI) (Shenzhen, China). The shotgun reads were assembled using the SOAPdenovo alignment tool (http://soap.genomics.org.cn/index.html#intro2). The potential open reading frames (ORFs) that encode proteins of more than 30 amino acids were determined by BLASTP searches (6). Conserved domain analyses of the translated ORFs were carried out using Batch CD-Search against the NCBI Conserved Domain Database.

Genome analyses revealed that BMBtp2 contains 36,932 bp of linear double-stranded DNA with a G+C content of 37.79%, which is consistent with that of the host strain YBT-1765. Sixty-nine ORFs that encode proteins of more than 30 amino acids were predicted. The majority of genes (47 ORFs) are transcribed in one orientation, and the rest are transcribed in the opposite orientation. The modular organization of the BMBtp2 genome follows the conserved pattern found in bacteriophages of the *Siphoviridae* family, infecting low-GC-percentage Gram-positive bacteria: DNA packaging proteins (small and large terminase subunits); head structural components and assembly proteins (portal protein, major capsid protein); head-tail structural components and assembly proteins (head-tail adaptor); tail structural components and assembly proteins (tail tape measure protein and tail fiber protein); host lysis proteins (endolysin); lysogeny-lytic control module (repressor, antirepressor, and integrase); and DNA replication, recombination, and modification proteins (recombinase, DNA helical, transcriptional regulator) (7–8). Interestingly, no typical holin protein was predicted in the host cell lysis module. In the similarity analysis, BMBtp2 showed high sequence similarity (85%) to a previously reported *Bacillus cereus* bacteriophage, TP21-L, which to some degree demonstrates that the two species have the same origin (9).

### Nucleotide sequence accession number. 

The complete genome sequence of *Bacillus thuringiensis* bacteriophage BMBtp2 is available in GenBank under accession no. JX887877.
